# Erratum: Kamiński, M., Łoniewski, I., Marlicz, W. Global Internet Data on the Interest in Antibiotics and Probiotics Generated by Google Trends. *Antibiotics* 2019, *8(3)*, 147

**DOI:** 10.3390/antibiotics8040190

**Published:** 2019-10-22

**Authors:** Mikołaj Kamiński, Igor Łoniewski, Wojciech Marlicz

**Affiliations:** 1 Sanprobi Sp.z.o.o. sp. k., Ul. Kurza Stopka 5/C, 70-535 Szczecin, Poland; 2 Faculty of Medicine I, Poznan University of Medical Sciences, 60-780 Poznań, Poland; 3 Department of Biochemistry and Human Nutrition, Pomeranian Medical University, 70-204 Szczecin, Poland; sanprobi@sanprobi.pl; 4 Department of Gastroenterology, Pomeranian Medical University, 70-204 Szczecin, Poland; marlicz@hotmail.com

1. We have found the error in the order of authors’ names and surnames in the article recently published in *Antibiotics* [1]:

Kamiński Mikołaj ^1,2,^*, Łoniewski Igor ^3^, Marlicz Wojciech ^4^

Which should be corrected to the following order in this paper [1]:

Mikołaj Kamiński ^1,2,^*, Igor Łoniewski ^3^ and Wojciech Marlicz ^4^

The authors would like to apologize for any inconvenience caused to the readers by these changes.
