# Supplementary figures and images for: Global Internet Data on the Interest in Antibiotics and Probiotics Generated by Google Trends
Source: Antibiotics (Basel). 2019 Sep 12;8(3):147. doi: 10.3390/antibiotics8030147 (PMC6784173; doi:10.3390/antibiotics8030147)

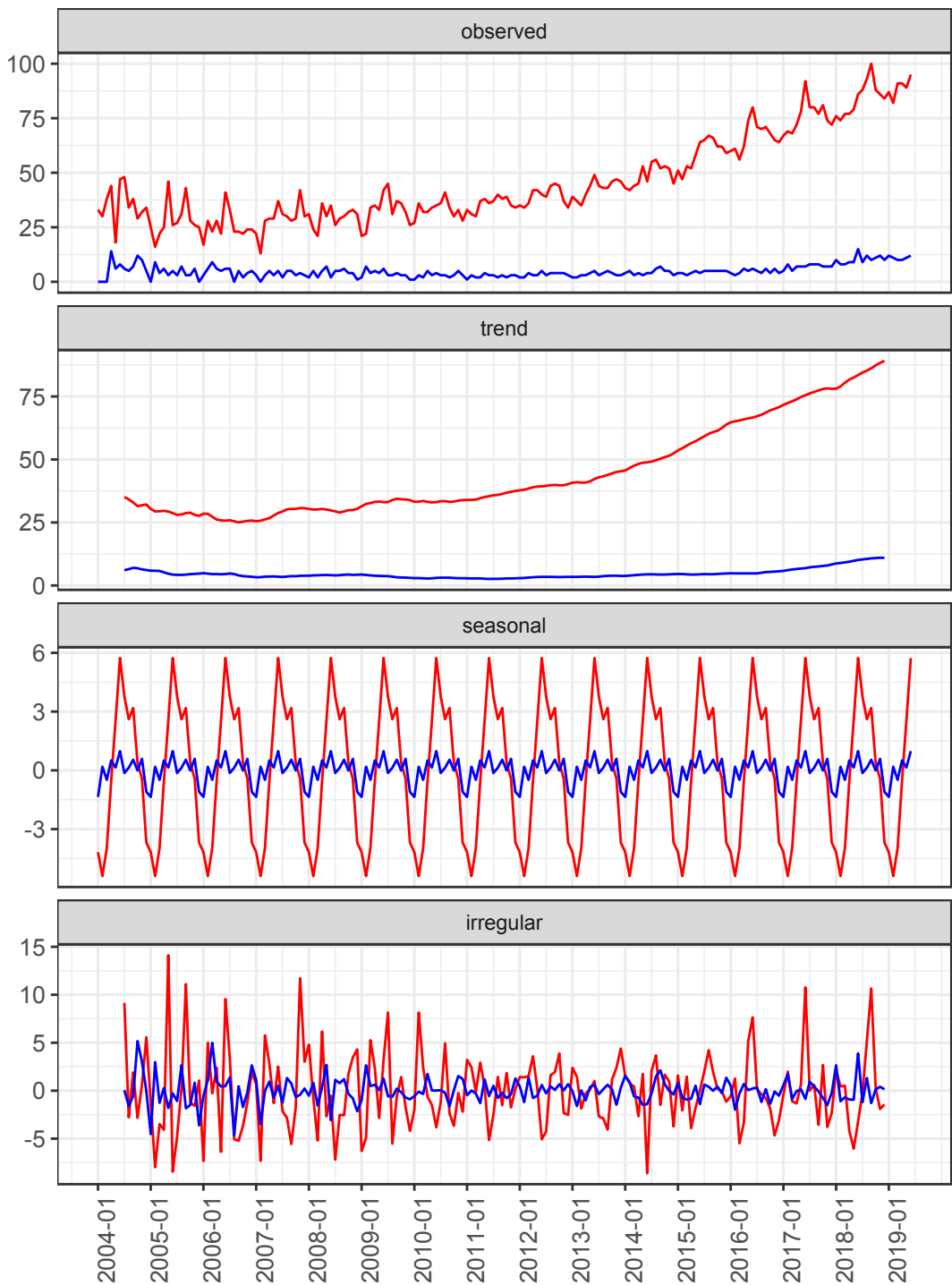

Keyword topic — Antibiotic — Probiotic

Supplement: Supplementary file 1 [file antibiotics-08-00147-s001.zip › Figure S2A.pdf]

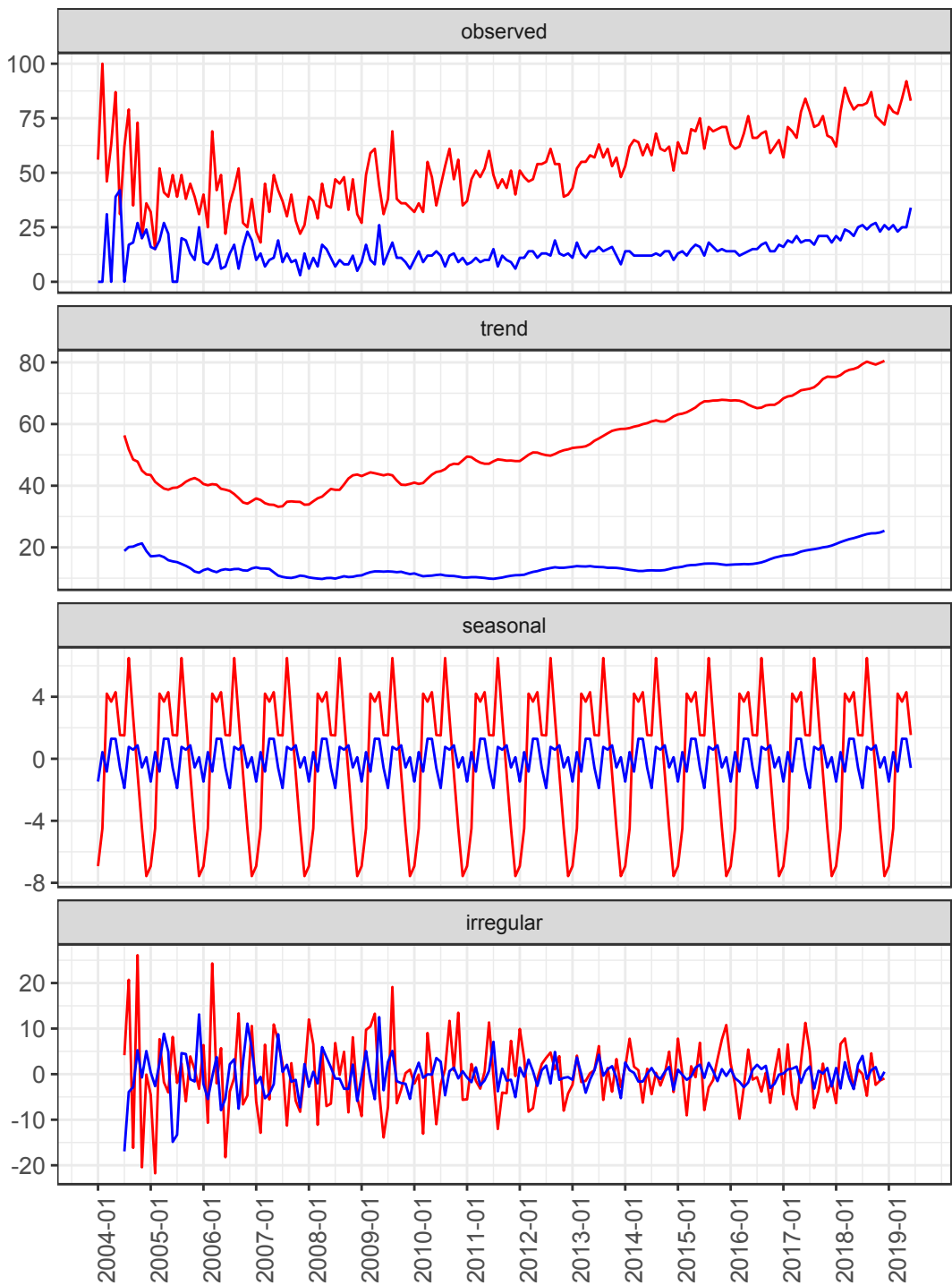

Keyword topic — Antibiotic — Probiotic

Supplement: Supplementary file 1 [file antibiotics-08-00147-s001.zip › Figure S2B.pdf]

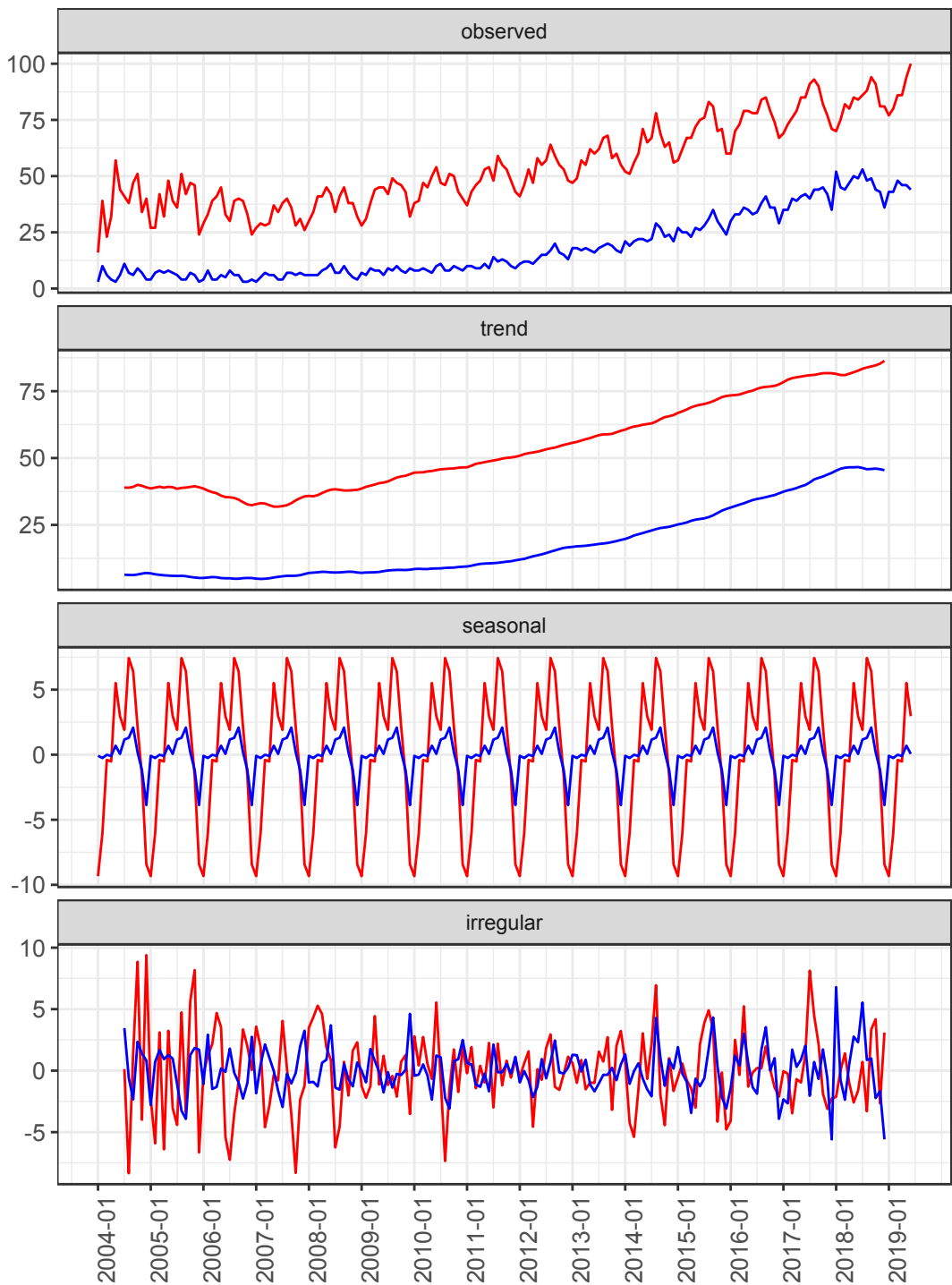

Keyword topic — Antibiotic — Probiotic

Supplement: Supplementary file 1 [file antibiotics-08-00147-s001.zip › Figure S2C.pdf]

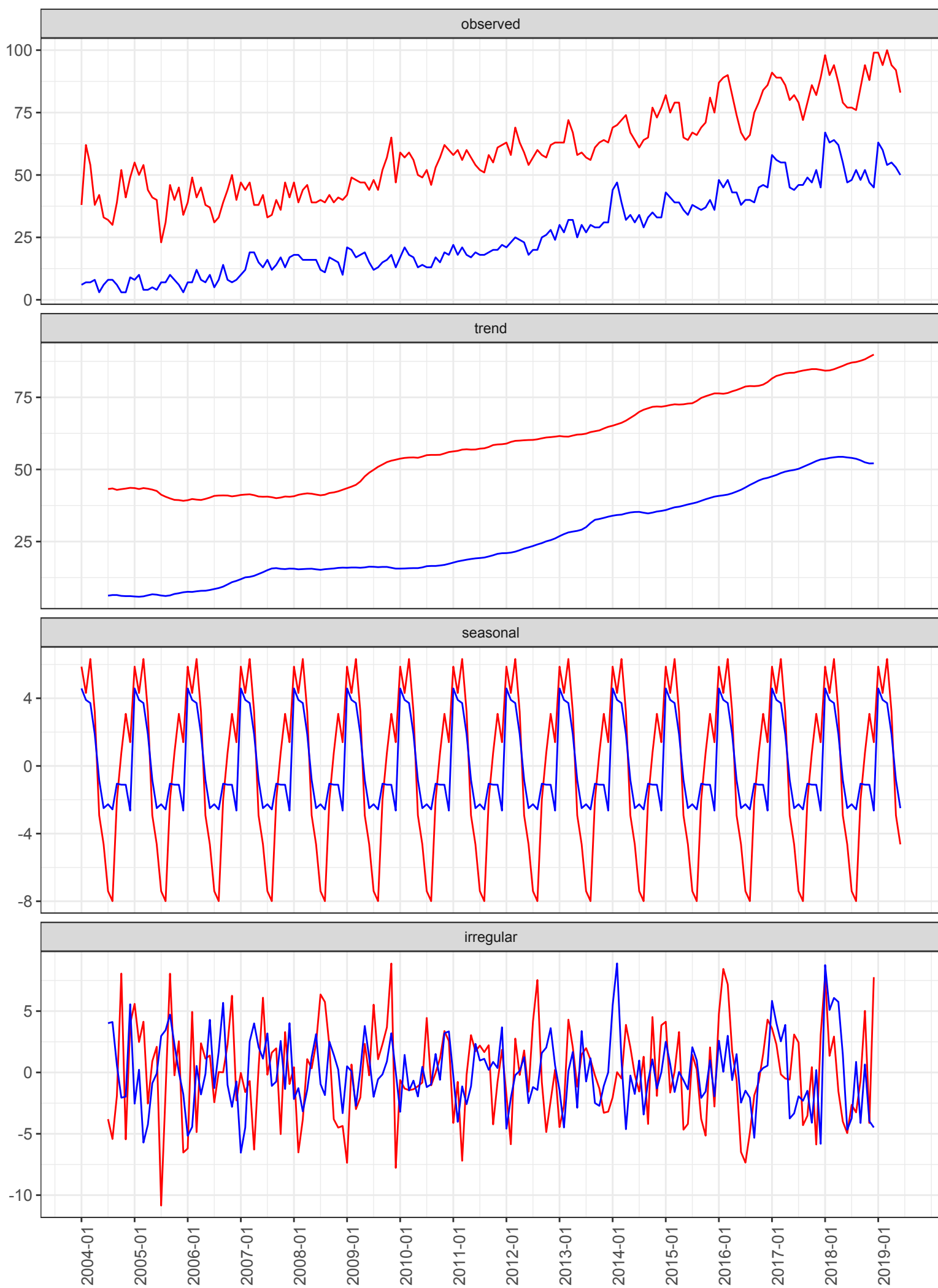

Keyword topic — Antibiotic — Probiotic

Supplement: Supplementary file 1 [file antibiotics-08-00147-s001.zip › Figure S3A.pdf]

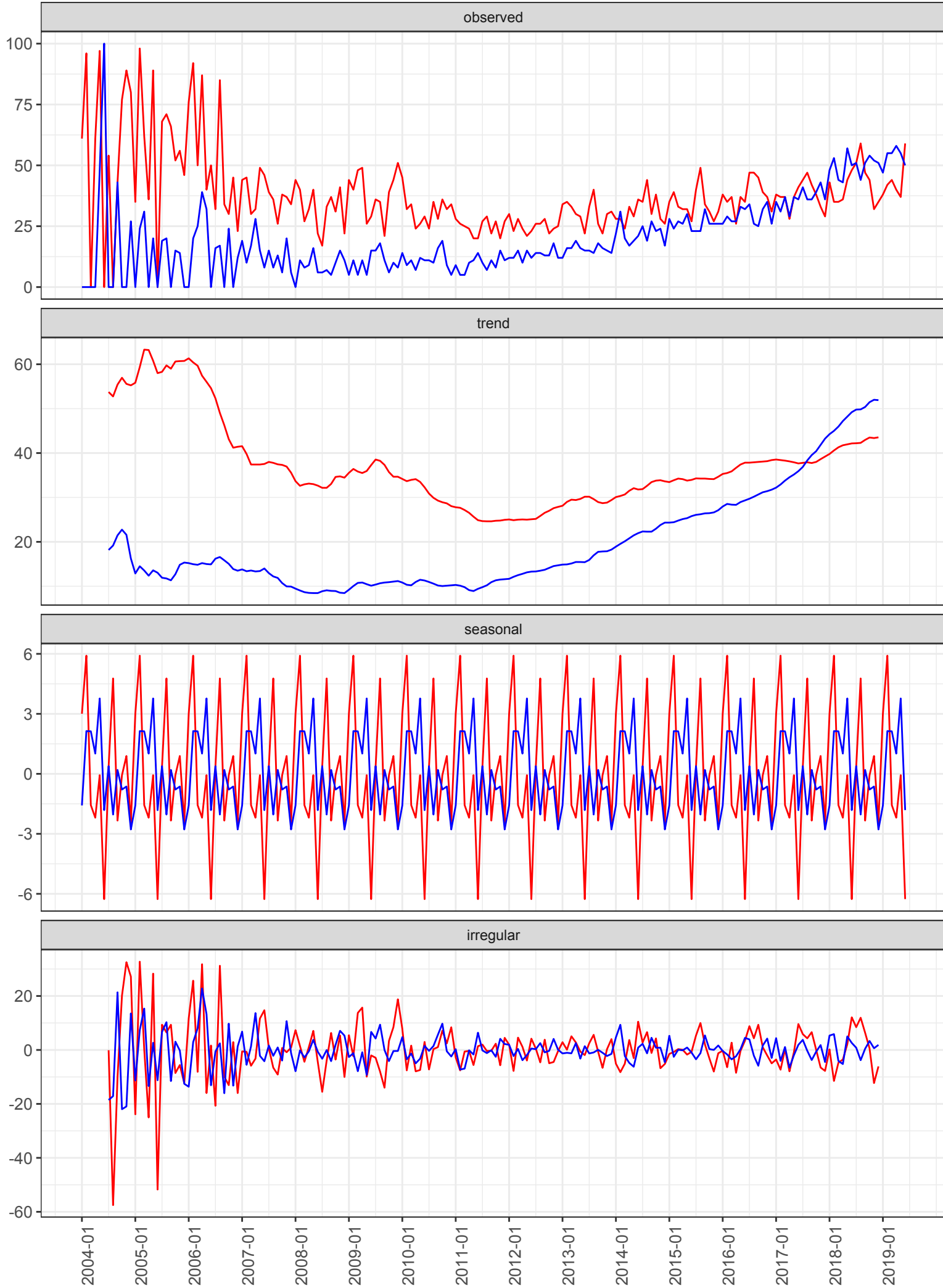

Keyword topic — Antibiotic — Probiotic

Supplement: Supplementary file 1 [file antibiotics-08-00147-s001.zip › Figure S3B.pdf]

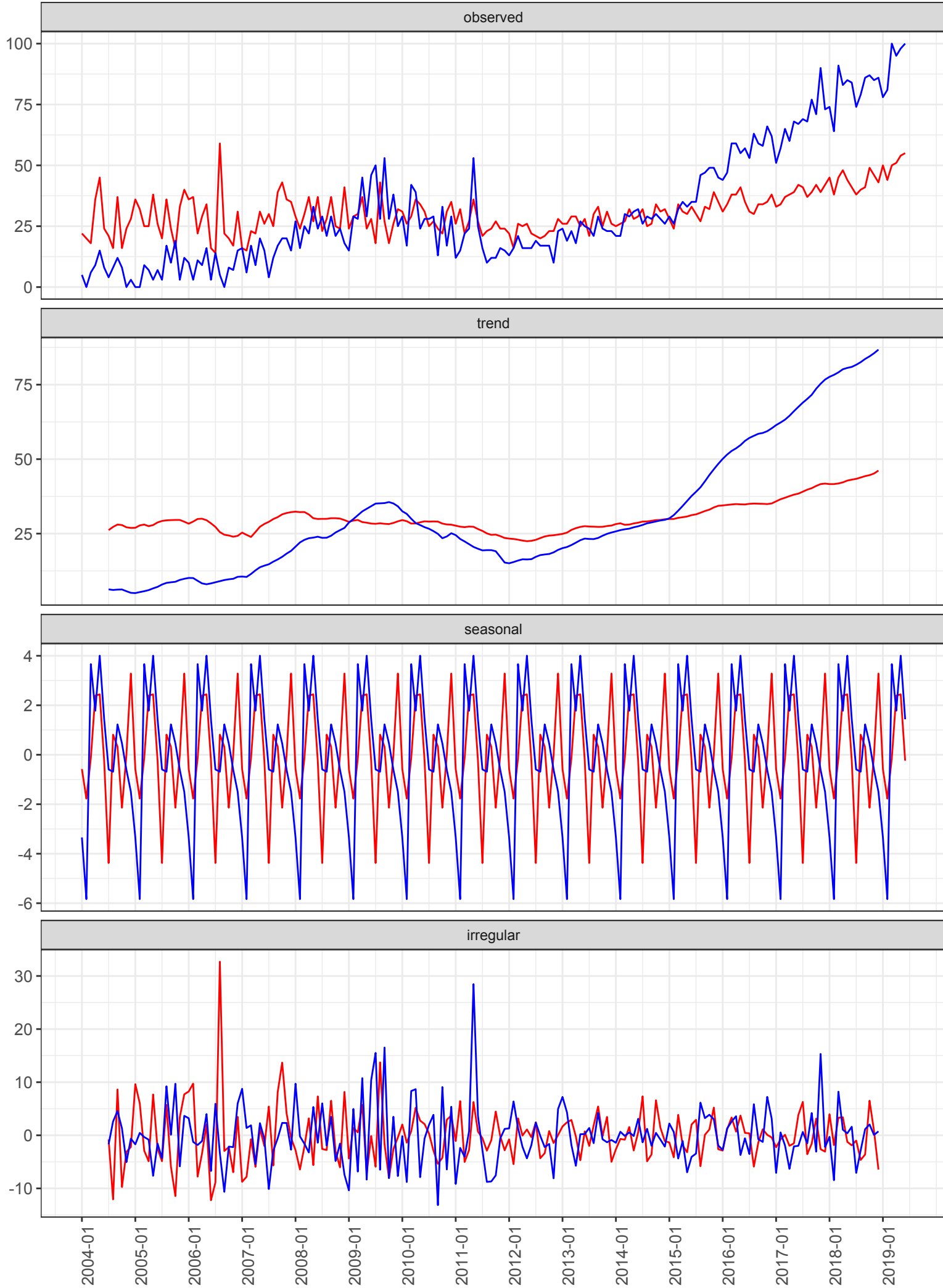

Keyword topic — Antibiotic — Probiotic

Supplement: Supplementary file 1 [file antibiotics-08-00147-s001.zip › Figure S3C.pdf]

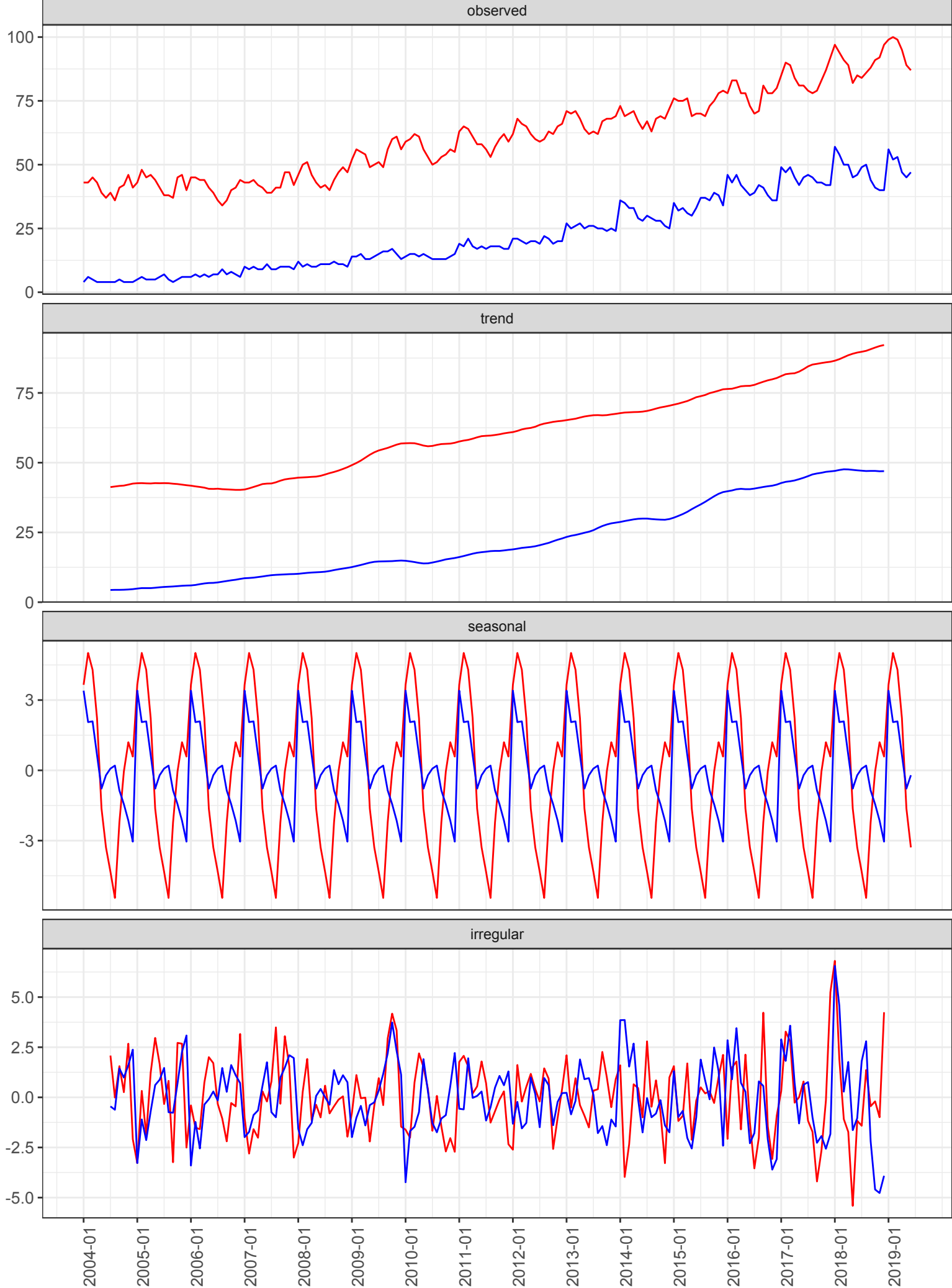

Keyword topic — Antibiotic — Probiotic

Supplement: Supplementary file 1 [file antibiotics-08-00147-s001.zip › Figure S3D.pdf]

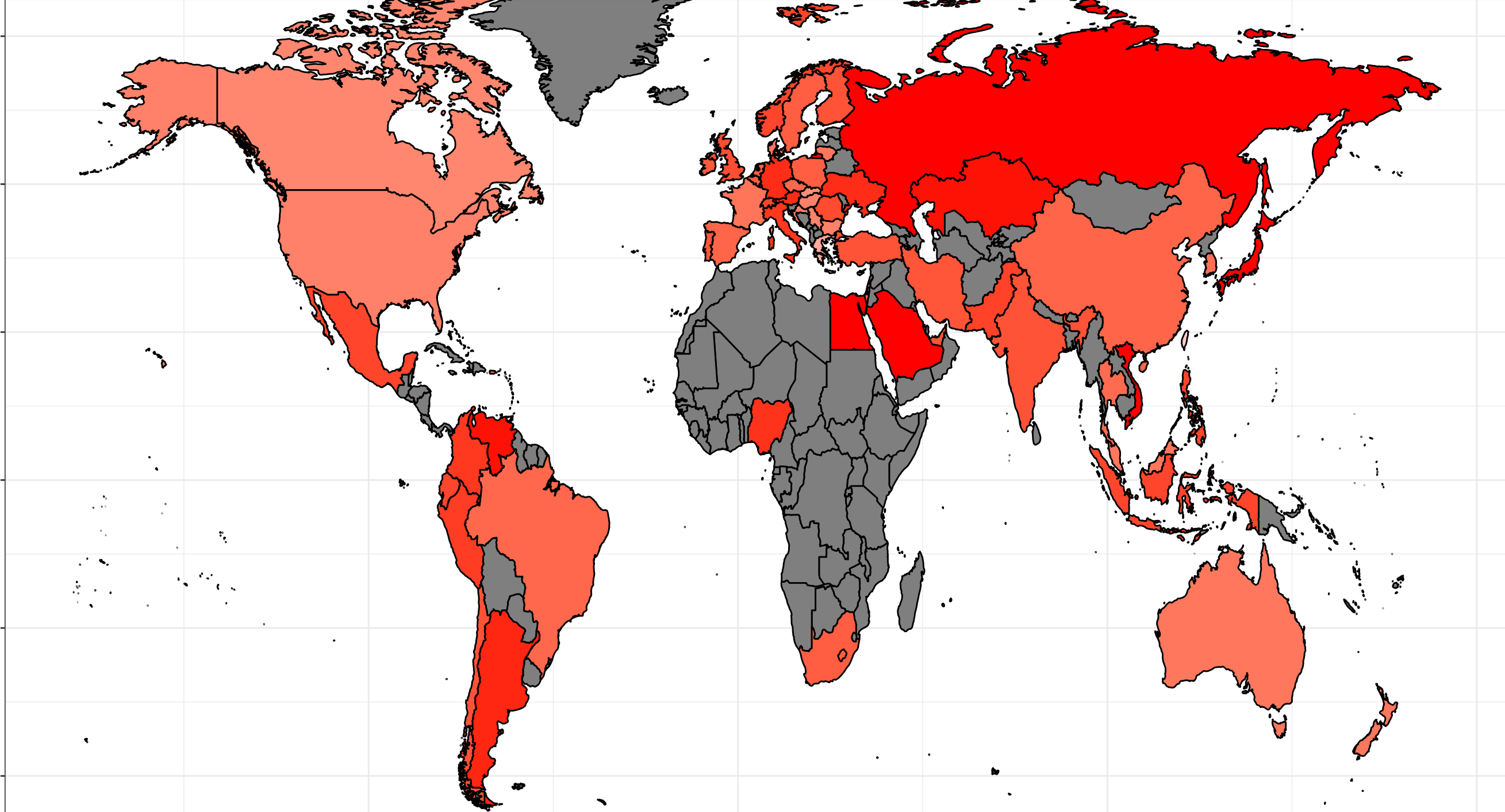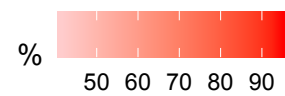

Supplement: Supplementary file 1 [file antibiotics-08-00147-s001.zip › Figure S1.pdf]
