# Supplementary material for: Global Internet Data on the Interest in Antibiotics and Probiotics Generated by Google Trends
Source: Antibiotics (Basel). 2019 Sep 12;8(3):147. doi: 10.3390/antibiotics8030147 (PMC6784173; doi:10.3390/antibiotics8030147)
Supplement: Supplementary file 1 [file antibiotics-08-00147-s001.zip › Table S1.doc]

Supplementary 1.

Checklist for Documentation of Google Trends research. Modified from Nuti et al.[10].

| **Section/Topic** | **Checklist item** |
| --- | --- |
| **Search Variables** |  |
| Access Date | 07 June 2019 |
| Time Period | From January 2004 to date of the collection (07 June 2019). |
| Query Category | All query categories were used |
| Region | Worldwide |
| Countries with Low Search Volume | Excluded |
| **Search Input** |  |
| Initial | Topics: „antibiotics”, „probiotic”, „antimicrobial resistance”, „prebiotic”, „synbiotics”. |
| The Secondary Input | Topics: with at least one Relative Search Volume > 10: „antibiotics”, „probiotic”. |
| **Rationale for Search Strategy** |  |
| For Search Input | The searched topics are related to treatment of bacterial infection and reconstitution of microbiota. |
| For Setting Chosen | We chose the abovementioned categories to not limit the output. |

10. S.V. Nuti, B. Wayda, I. Ranasinghe, S. Wang, R.P. Dreyer, S.I. Chen, K. Murugiah, The Use of Google Trends in Health Care Research: A Systematic Review, PLoS ONE. 9 (2014) e109583. doi:10.1371/journal.pone.0109583.
