# Supplementary material for: Global Internet Data on the Interest in Antibiotics and Probiotics Generated by Google Trends
Source: Antibiotics (Basel). 2019 Sep 12;8(3):147. doi: 10.3390/antibiotics8030147 (PMC6784173; doi:10.3390/antibiotics8030147)
Supplement: Supplementary file 1 [file antibiotics-08-00147-s001.zip › Table S2.doc]

Table S2.

Additional the time series analysis of the topics. Relative Search Volume presented as mean ± standard deviation.

* p < 0.05, ** p < 0.01, *** p < 0.001

| Topic | Location | RSV | Seasonal Mann-Kendall test | Slope | TBATS (seasonality present, period [month]) | Month with the lowest seasonal component | Month with the highest seasonal component | Seasonal component amplitude |
| --- | --- | --- | --- | --- | --- | --- | --- | --- |
| Antibiotics | Canada | 59.9 ± 17.9 | tau = 0.86*** | 3.5 RSV/year  (6.0%/year) | YES, 12 | August  (-8.0 RSV) | March  (6.3 RSV) | 13.3 RSV  (23.9%) |
| Probiotic | Canada | 26.1 ± 16.34 | tau = 0.91*** | 3.4 RSV/year  (13.2%/year) | YES, 12 | August  (-2.6 RSV) | January  (4.6 RSV) | 7.2 RSV  (27.7%) |
| Antibiotics | Greece | 38.1 ± 16.8 | tau = -0.05; NS | - | NO, - | - | - | - |
| Probiotic | Greece | 20.5 ± 15.7 | tau = 0.59*** | 2.2 RSV/year  (10.5 %/year) | NO, - | - | - | - |
| Antibiotics | Taiwan | 30.8 ± 8.6 | tau = 0.42*** | 1.0 RSV/year  (3.1%/year) | YES, 12 | July  (-4.4 RSV) | December  (3.3 RSV) | 7.7 RSV  (24.9%) |
| Probiotic | Taiwan | 32.3 ± 25.0 | tau = 0.74*** | 4.7 RSV/year  (14.4%) | YES, 12 | February  (-5.8 RSV) | May  (4.0 RSV) | 9.8 RSV  (30.5%) |
| Antibiotics | The United States | 61.5 ± 17.0 | tau = 0.93*** | 3.6 RSV/year  (5.9%) | YES, 12 | August  (-5.4 RSV) | February  (5.0 RSV) | 10.5 RSV  (17.0%) |
| Probiotic | The United States | 22.7 ± 14.9 | tau = 0.97*** | 3.2 RSV/year  (14.1%) | YES, 12 | December  (-3.0 RSV) | January  (3.4 RSV) | 6.4 RSV  (28.4%) |

NS – Non significant; RSV – Relative Search Volume, TBATS - exponential smoothing state space model with Box-Cox transformation, autoregressive-moving average errors, and trend and seasonal components
